# A biomarker framework for auditory system aging: the Aging Biomarker Consortium consensus statement

**DOI:** 10.1093/lifemedi/lnaf011

**Published:** 2025-03-07

**Authors:** Aging Biomarker Consortium, Xiaolong Fu, Si Wang, Yunhao Wu, Yu Sun, Wenwen Liu, Xin Xi, Geng-Lin Li, Ke Liu, Wei Yuan, Fangyi Chen, Hongyang Wang, Tao Yang, Yuhe Liu, Jialin Zheng, Haibo Shi, Jing Qu, Xiaowei Chen, Limin Suo, Yideng Huang, Xinbo Xu, Xuxia Tang, Xiaojun Li, Lei Xu, Xia Gao, Lisheng Yu, Yilai Shu, Weiqi Zhang, Jinpeng Sun, Huijun Yuan, Shusheng Gong, Wenyan Li, Xiulan Ma, Dingjun Zha, Jiangang Gao, Huawei Li, Zuhong He, Guang-Hui Liu, Gang Pei, Weijia Kong, Haibo Wang, Renjie Chai

**Affiliations:** Shandong Provincial Hospital, Medical Science and Technology Innovation Center, School of Clinical and Basic Medical Sciences, Shandong First Medical University & Shandong Academy of Medical Sciences, Jinan 250117, China; Shandong Provincial Hospital, Medical Science and Technology Innovation Center, School of Clinical and Basic Medical Sciences, Shandong First Medical University & Shandong Academy of Medical Sciences, Jinan 250117, China; Advanced Innovation Center for Human Brain Protection, National Clinical Research Center for Geriatric Disorders, Xuanwu Hospital Capital Medical University, Beijing 100053, China; Aging Translational Medicine Center, Beijing Municipal Geriatric Medical Research Center, Xuanwu Hospital, Capital Medical University, Beijing 100053, China; Shandong Provincial Hospital, Medical Science and Technology Innovation Center, School of Clinical and Basic Medical Sciences, Shandong First Medical University & Shandong Academy of Medical Sciences, Jinan 250117, China; Department of Otorhinolaryngology, Union Hospital, Tongji Medical College, Huazhong University of Science and Technology, Wuhan 430074, China; Department of Otolaryngology-Head and Neck Surgery, Shandong Provincial ENT Hospital, Shandong University, Jinan 250022, China; Department of Otolaryngology Head and Neck Surgery, Chinese PLA General Hospital, Beijing 100000, China; ENT Institute and Department of Otorhinolaryngology, Eye and ENT Hospital, Fudan University, Shanghai 200433, China; Department of Otolaryngology Head and Neck Surgery, Beijing Friendship Hospital of Capital Medical University, Beijing 100050, China; Department of Otolaryngology & Head and Neck, Chongqing General Hospital, Chongqing 401147, China; Department of Biology, South University of Science and Technology of China, Shenzhen 518000, China; Senior Department of Otolaryngology Head and Neck Surgery, the sixth Medical Center of Chinese PLA General Hospital, Medical School of Chinese PLA, Beijing 100853, China; State Key Laboratory of Hearing and Balance Science, Beijing 100853, China; National Clinical Research Center for Otolaryngologic Diseases, Beijing 100853, China; Department of Otolaryngology-Head and Neck Surgery, Shanghai Ninth People’s Hospital, Shanghai Jiaotong University School of Medicine, Shanghai 200023, China; Department of Otolaryngology, Head and Neck Surgery, Beijing Friendship Hospital, Capital Medical University, Beijing 100050, China; Collaborative Innovation Center for Brain Science, Tongji University, Shanghai 200092, China; Department of Otolaryngology Head & Neck Surgery, Shanghai Jiao Tong University Affiliated Sixth People’s Hospital, Shanghai 200233, China; State Key Laboratory of Stem Cell and Reproductive Biology, Institute of Zoology, Institute for Stem Cell and Regeneration, Institute for Stem Cell and Regenerative Medicine, University of Chinese Academy of Sciences, Beijing 100101, China; Department of Otolaryngology, Peking Union Medical College Hospital, Beijing 100730, China; Department of Otolaryngology, Head and Neck Surgery, the Second Hospital of Shanxi Medical University, Taiyuan 030001, China; Department of Otolaryngology, The First Affiliated Hospital of Wenzhou Medical University, Wenzhou 325000, China; Department of Otolaryngology, Qilu Hospital of Shandong University, Jinan 250012, China; Otolaryngology Department, Zhejiang Provincial Hospital of TCM, Hangzhou 310003, China; Frontier Institute of Science and Technology, Xi’an Jiaotong University, Xi’an 710049, China; Department of Otolaryngology-Head and Neck Surgery, Shandong Provincial ENT Hospital, Shandong University, Jinan 250022, China; Department of Otolaryngology-Head and Neck Surgery, the Affiliated Drum Tower Hospital of Nanjing University Medical School, Jiangsu Provincial Key Medical Discipline (Laboratory), Nanjing 210008, China; Department of Otolaryngology, Head and Neck Surgery, People’s Hospital, Peking University, Beijing 100044, China; ENT Institute and Department of Otorhinolaryngology, Eye & ENT Hospital, State Key Laboratory of Medical Neurobiology and MOE Frontiers Center for Brain Science, MOE Engineering Research Center of Gene Technology, Fudan University, Shanghai 200031, China; China National Center for Bioinformation, Beijing 100101, China; Beijing Institute of Genomics, Chinese Academy of Sciences, Beijing 100101, China; NHC Key Laboratory of Otorhinolaryngology, Qilu hospital and School of Basic Medical Sciences, Shandong University, Jinan 250012, China; Department of Oto-Rhino-Laryngology, West China Hospital, Sichuan University, Chengdu 610000, China; Department of Otolaryngology-Head and Neck Surgery, Beijing Friendship Hospital, Capital Medical University, Beijing 100050, China; ENT institute and Otorhinolaryngology Department of Eye & ENT Hospital, State Key Laboratory of Medical Neurobiology and MOE Frontiers Center for Brain Science, Fudan University, Shanghai 200031, China; Department of Otolaryngology Head and Neck Surgery, Shengjing Hospital of China Medical University, Shenyang 110000, China; Department of Otolaryngology Head and Neck Surgery, Xijing Hospital, Fourth Military Medical University, Xi’an 710000, China; School of Life Science and Key Laboratory of the Ministry of Education for Experimental Teratology, Shandong University, Jinan 250100, China; ENT institute and Otorhinolaryngology Department of Eye & ENT Hospital, State Key Laboratory of Medical Neurobiology and MOE Frontiers Center for Brain Science, Fudan University, Shanghai 200031, China; Department of Otorhinolaryngology‑Head and Neck Surgery, Zhongnan Hospital of Wuhan University, Wuhan 430071, China; State Key Laboratory of Membrane Biology, Institute of Zoology, Institute for Stem Cell and Regeneration, University of Chinese Academy of Sciences, Chinese Academy of Sciences, Beijing 100101, China; Collaborative Innovation Center for Brain Science, School of Life Science and Technology, Tongji University, Shanghai 200092, China; Department of Otorhinolaryngology, Union Hospital, Tongji Medical College, Huazhong University of Science and Technology, Wuhan 430030, China; Department of Otolaryngology-Head and Neck Surgery, Shandong Provincial ENT Hospital, Shandong University, Jinan 250022, China; State Key Laboratory of Digital Medical Engineering, Department of Otolaryngology Head and Neck Surgery, Zhongda Hospital, School of Life Sciences and Technology, Advanced Institute for Life and Health, Jiangsu Province High-Tech Key Laboratory for Bio-Medical Research, Southeast University, Nanjing 210096, China; Co-Innovation Center of Neuroregeneration, Nantong University, Nantong 226001, China; School of medical technology, Institute of Engineering Medicine, Beijing Institute of Technology, Beijing 100081, China

## Abstract

Hearing is one of the most vital sensory functions in human beings and a crucial means of perceiving and acquiring information from the natural environment. The advancement of human society is closely linked to the development of language, with hearing serving as the foundation for verbal communication. As individuals age, the deterioration of the auditory system becomes a significant factor contributing to sensory impairments in the elderly. In addition to hearing loss, the aging of the auditory system is also associated with cognitive decline and psychosocial disorders, which severely impact the quality of life for older adults. Currently, there are no effective treatments or interventions available for addressing the aging of the auditory system. Therefore, identifying biomarkers of the auditory system aging is of great significance. The Aging Biomarker Consortium of China has conducted a comprehensive evaluation of aging biomarkers in the auditory system, focusing on three dimensions: morphological, functional, and humoral biomarkers. This initiative aims to establish a foundation for assessing the degree of aging in the auditory system and to promote the management of auditory health in an aging society, ultimately enhancing the auditory health of the elderly population both in China and globally.

## Introduction

The auditory system is primarily composed of two components: the peripheral auditory system and the central auditory system [1, 2]. The peripheral auditory system encompasses the outer ear, middle ear, and inner ear. The outer ear consists of the auricle and the external auditory canal. The middle ear includes the nasopharynx and the eustachian tube (located anteriorly), as well as the mastoid air cells (located posteriorly). The inner ear primarily comprises the cochlea, which processes sound information, and the vestibular organ, which detects positional information [3]. The central auditory system extends through the cerebral cortex, brainstem, midbrain, and thalamus, representing one of the longest central pathways within the sensory system. Key nuclei of the central auditory system include the cochlear nucleus, superior olivary complex, lateral lemniscus, inferior colliculus, and medial geniculate body.

The cochlea is a critical component of the auditory system, responsible for converting sound wave vibrations into electrical signals that are transmitted to the brain, thereby facilitating hearing [4]. As individuals age, the physiological function of the cochlea gradually declines, leading to presbycusis, which adversely affects the quality of life in the elderly, manifesting as communication disorders, social isolation, and psychological issues. Cochlear senescence is a significant contributor to age-related hearing loss. The aging mechanisms of the cochlea are complex, involving various anatomical regions, including the organ of Corti, modiolus, stria vascularis, spiral ligament, and numerous tissue cell types [5, 6]. Characteristics of cochlear aging include the loss of essential cell types, such as inner hair cells, outer hair cells, spiral neurons, and fibroblasts in the spiral ligament area, along with age-related atrophy of the stria vascularis. Therefore, assessing this aging process is vital for the early detection of hearing loss and for implementing timely interventions and treatments to mitigate the progression of hearing loss.

With the increasing aging of the population, promoting healthy aging has become an important social goal. Therefore, the assessment of auditory system aging is of great significance for understanding the mechanisms of hearing loss, improving the quality of life for the elderly, preventing related diseases, and developing new remedies. By evaluating the aging of the auditory system, we can assist the elderly in maintaining good auditory function. Preventive measures, such as avoiding noise exposure and enhancing lifestyle habits, can be implemented to reduce the risk of hearing disabilities. On 16 November 2024, the Chinese Aging Biomarker Consortium (ABC) convened a symposium in Jinan, bringing together experts in the field of auditory system aging. Drawing on literature reports and both domestic and international peer research, along with evidence-based medical findings and perspectives that reflect both global and Chinese characteristics, a consensus on the biological biomarkers of aging in the auditory system has been established.

## Recommended methodology for biomarkers for auditory system aging

The levels of recommendation and evidence in this consensus are presented in a manner that is internationally recognized [7], as illustrated in Table 1. All recommendations underwent a thorough review and discussion among the members of the ABC, facilitating the incorporation of diverse perspectives and considerations into this consensus.

**Table 1.** Class of recommendations and level of evidence

CLASS (STRENGTH) OF RECOMMENDATION	LEVEL (QUALITY) OF EVIDENCE
**CLASS I (STRONG) Benefit >>> Risk**	**Level A**
**Suggested phrases for writing recommendation** • Recommendation/indicated • Evidence and/or general agreement that a given treatment or procedure is beneficial, useful and effective	• Data derived from multiple randomized clinical trials or meta-analyses
**CLASS Ila (MODERATE) Benefit >> Risk**	**Level B**
**Suggested phrases for writing recommendation** • Should be considered • Weight of evidence/opinion is in favor of usefulness/efficacy	• Data derived from a single randomized clinical trial or large non-randomized studies
**CLASS IIb (WEAK) Benefit ≥ Risk**	**Level C**
**Suggested phrases for writing recommendation** • May be considered • Usefulness/efficacy is less well established by evidence/opinion	• Consensus of expert opinion, and/or small studies, retrospective studies, registries
**Class III (STRONG) Risk > Benefit**	**Note:** COR and LOE are determined independently (any COR may be paired with any LOE). COR, Class of Recommendation; LOE, Level of Evidence.
**Suggested phrases for writing recommendation** Not recommended • Evidence/general agreement that the given treatment/procedure is not useful/effective and sometimes maybe harmful	

## Classification and clinical application of auditory system aging biomarkers

**Figure 1.**
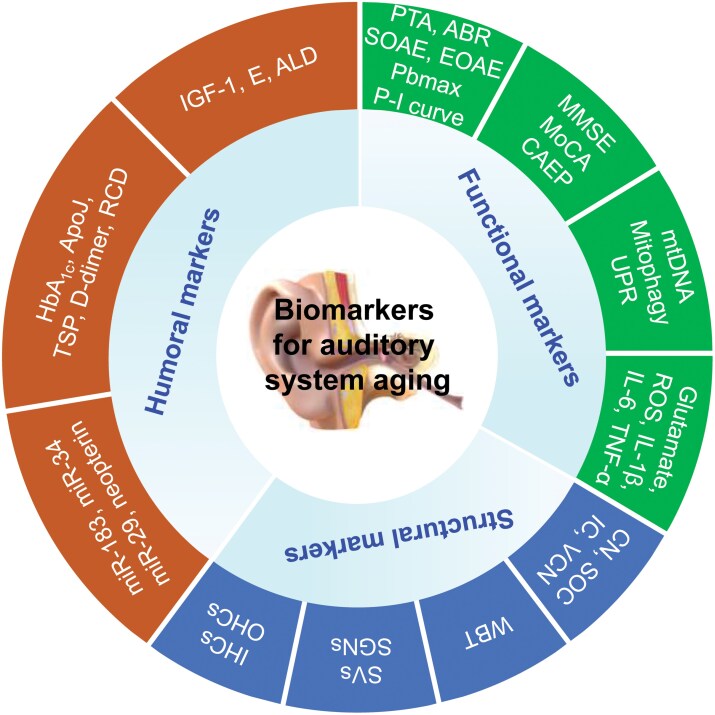
**Framework of biomarkers for auditory system aging.** The proposed framework for auditory system aging consists of three dimensions: functional, structural, and humoral biomarkers. The recommended biomarkers cover multi-dimensional and multi-hierarchical changes in the auditory system aging. Abbreviations: ABR, auditory brainstem response; ALD, aldosterone; ApoJ, apolipoprotein J; CAEP, cortical auditory evoked potential; CN, cochlear nucleus; E, estrogen; EOAE, evoked otoacoustic emission; HbA_1c_, glycosylated hemoglobin; IC, inferior colliculus; IGF-1, insulin-like growth factor 1; IHCs, inner hair cells; IL-1β, interleukin 1β; MMSE, mini mental status exam; MoCA, montreal cognitive assessment; mtDNA, mitochondrial DNA; OHCs, outer hair cells; Pbmax, maximum speech recognition score; PTA, pure tone audiometry; RCD, red blood cell deformability; ROS, reactive oxygen species; SGNs, spiral ganglion cells; SOAE, spontaneous otoacoustic emission; SOC, superior olivary complex; SVs, stria vascularis; TNF-α, tumor necrosis factor α; TSP, thrombospondin; UPR, unfolded protein response; VCN, ventral cochlear nucleus; WBT, wideband tympanometry test.

The aging of the auditory system involves multi-dimensional and multi-hierarchical changes at the molecular, cellular, organ, and organismal levels. Biomarkers of auditory system aging are defined as indicators that can accurately predict the “true age” of the auditory system, alongside its “structure” and “function”. These biomarkers are essential for evaluating both the degree and rate of aging within the auditory system, as well as the associated risk of disease and the impact of aging interventions. This consensus identifies biomarkers of auditory system aging across three dimensions: function, structure, and body fluids (Fig. 1) for reference in clinical practice and future research.

### Functional markers of auditory system aging

Aging of the auditory system is primarily manifested as presbycusis, or age-related hearing loss. This condition has emerged as the third most prevalent chronic disease among the elderly population. Reports indicate that the incidence rate of age-related hearing loss increases exponentially with age, reaching 25% among individuals over 65 years, 75% among those over 75 years, and 99% among centenarians. Hearing function declines progressively with age, with the rate of hearing loss in the better ear estimated at 5.5 to 8 dB every decade, while the deterioration in the worse ear occurs at a faster rate. Notably, there are gender differences in both the rate and incidence of hearing loss, with males being more frequently affected; however, by the age of 80 to 90, the disparity between genders becomes negligible. The aging auditory system significantly impacts the quality of life for the elderly, leading to communication barriers, sensory deprivation, and subsequent cognitive changes, personality alterations, social isolation, and other adverse effects.

#### Decreased hearing function

##### Loss of high-frequency hearing

Humans and animals, such as mice, often exhibit high-frequency hearing loss as they age, which serves as a significant indicator of auditory system aging. Reports indicate that C57BL/6J mice displayed high-frequency hearing loss in auditory function tests conducted at 3, 6, and 15 months of age, mirroring the characteristics of human presbycusis [8]. Pure tone audiometry (PTA) is one of the fundamental methods for evaluating hearing function (Table 2) [9]. This test assesses hearing status by detecting the minimum audible intensity of sounds across various frequencies. Elderly individuals typically exhibit more pronounced hearing loss in the high-frequency range.

**Table 2.** Grades of hearing loss and related hearing experience by WHO in 2021

Grade	Hearing threshold in better hearing ear in decibels (dB)	Hearing experience in a quiet environment for most adults	Hearing experience in a noisy environment for most adults
**Normal hearing**	Less than 20 dB	No problem hearing sounds	No or minimal problem hearing sounds
**Mild hearing loss**	20 to < 35 dB	Does not have problems hearing conversational speech	May have difficulty hearing conversational speech
**Moderate hearing loss**	35 to < 50 dB	May have difficulty hearing conversational speech	Difficulty hearing and taking part in conversation
**Moderately severe hearing loss**	50 to < 65 dB	Difficulty hearing conversational speech; can hear raised voices without difficulty	Difficulty hearing most speech and taking part in conversation
**Severe hearing loss**	65 to < 80 dB	Does not hear most conversational speech; may have difficulty hearing and understanding raised voices	Extreme difficulty hearing speech and taking part in conversation
**Profound hearing loss**	80 to < 95 dB	Extreme difficulty hearing raised voices	Conversational speech cannot be heard
**Complete or total hearing loss/deafness**	95 dB or greater	Cannot hear speech and most environmental sounds	Cannot hear speech and most environmental sounds
**Unilateral**	< 20 dB in the better ear, 35 dB or greater in the worse ear	May not have problem unless sound is near the poorer hearing ear. May have difficulty in locating sounds	May have difficulty hearing speech and taking part in conversation, and in locating sounds

##### Decline of the ability of speech recognition

Even in relatively quiet environments, elderly individuals may struggle to comprehend conversations, particularly in the presence of background noise. This difficulty reflects a decline in the cochlea’s and the brain’s ability to process auditory information. The maximum speech recognition rate (Pbmax) indicates the highest rate of speech recognition achievable under optimal listening conditions for patients with hearing impairments, thereby providing insight into their speech recognition capabilities in daily communication [10]. Notably, there is a significant correlation between the cognitive function of elderly patients with hearing loss and their maximum speech recognition rate.

To assess speech recognition rates at varying speech intensity levels, multiple equivalent tests can be administered sequentially. This approach allows for the construction of the function curve of speech recognition rate and intensity (PI curve) for hearing-impaired patients, facilitating the estimation of the dynamic range of speech intensity that corresponds to speech recognition rates of 20%–80%.

##### Decreased or disappeared otoacoustic emission

Otoacoustic emissions (OAE) are weak sound signals that are spontaneously generated when the cochlea is stimulated [11]. In healthy individuals, these signals can be measured; however, in certain types of age-related hearing loss, OAE may be diminished or completely absent.

##### Abnormity of auditory brainstem response

The auditory brainstem response (ABR) measurement is a technique used to objectively assess the functional status of the auditory pathway, spanning from the cochlea to the brainstem [12]. In patients experiencing age-related hearing loss, the auditory brainstem response may exhibit characteristics such as prolonged latency and alterations in waveform.

#### Decreased cognitive function

The Mini-Mental Status Examination (MMSE) is the most widely utilized screening tool for cognitive impairment, both domestically and internationally [13]. It assesses various cognitive domains, including orientation, memory, attention, calculation, language ability, and visuospatial skills. The MMSE can be employed to evaluate the cognitive function of patients with presbycusis. However, the scores obtained from this examination are influenced by factors such as age and educational background. Elderly individuals with higher education levels may experience false negatives, while those with lower education levels may encounter false positives. Consequently, this method is not recommended for comprehensive cognitive assessment.

The Montreal Cognitive Assessment (MoCA) is a tool designed for the rapid screening of cognitive dysfunction. Its advantages include higher sensitivity for detecting mild cognitive impairment, a broader and more comprehensive coverage of cognitive domains, and a more nuanced assessment of memory [14]. However, the MoCA scale is influenced by factors, such as the examinee’s educational level, cultural background, the examiner’s skill and experience in administering the MoCA, the testing environment, and the emotional and mental state of the examinee. Consequently, it is not recommended to use this method as an assessment indicator for cognitive function in patients with age-related hearing loss.

The cortical auditory evoked potential (CAEP) is an electrical potential generated by auditory stimulation within the central auditory system. It is recorded using multiple electrodes positioned at specific locations on the skull surface and can be utilized to evaluate the feasibility of speech perception abilities. As an electrophysiological test, CAEP offers several advantages, including objectivity, the capacity to be tested in a conscious state, the use of speech stimuli, and strong consistency with behavioral hearing thresholds [15]. Due to its sensitivity to the physical properties of the stimulus signal, the P1-N1-P2 complex wave is often referred to as the mandatory response, which reflects the brain’s passive sensory processing of sound signals [16]. Consequently, it holds significant clinical value in assessing speech recognition capabilities and cognitive function.

#### Changes of the internal cochlear environment

##### Changes of neurotransmitter

Glutamate serves as an excitatory synaptic transmitter in the central nervous system and functions as a neurotransmitter between the inner hair cells of the cochlea and the dendrites of the auditory nerve [17]. As aging progresses, the excessive release of glutamate directly or indirectly impacts the postsynaptic neuron receptors, resulting in an influx of ions that subsequently draws in a significant amount of water, culminating in acute dendritic edema. Concurrently, the influx of calcium ions can disrupt the homeostasis of the intracellular calcium environment, potentially leading to cell death and resulting in damage and dysfunction of inner ear cells [18]. This toxicity is also observed in acute injuries to the cochlear organ of Corti, which leads to edema of radial nerve fibers and the loss of type I neurons.

##### Accumulation of free radical

The accumulation of reactive oxygen species (ROS) in the cochlea typically accompanies caspase-mediated apoptosis [19]. The oxidative stress induced by ROS during cochlear aging leads to damage of spiral neurons and the stria vascularis. ROS, which are toxic byproducts of cellular metabolism derived from molecular oxygen, include various molecules and free radicals, such as hydroxyl radicals, superoxide anions, hydrogen peroxide, and singlet oxygen, predominantly produced by mitochondria within cells [20]. Free radicals are significant pathogenic factors in aging-related diseases, including age-related hearing loss. In the aging cochlea, the increase in ROS disrupts the homeostasis of the inner ear, resulting in hair cell loss and subsequent hearing impairment. Furthermore, during the aging process, the rise in ROS levels is accompanied by a gradual decline in the function of the antioxidant defense system. Consequently, endogenous enzymes (antioxidases) that protect cells from ROS damage are produced in reduced quantities or function abnormally. This imbalance between ROS production and antioxidant defense systems exacerbates oxidative stress and contributes to hearing loss [21].

##### Inflammation

Inflammation plays a crucial role in aging, as well as in drug- and noise-induced hearing loss. Inflammatory cells infiltrate and proliferate within the cochlea, where they can synthesize and release a variety of pro-inflammatory cytokines, including interleukin-1β (IL-1β), interleukin-6 (IL-6), and tumor necrosis factor-α (TNF-α) [22]. IL-1β and IL-6 serve as acute inflammatory markers that belong to the interleukin superfamily. These cytokines facilitate the infiltration of mononuclear phagocytes, which subsequently release elastase and prostaglandins. This process leads to the excessive activation of the inflammatory cascade, resulting in microcirculation disorders, tissue damage, and ultimately, hearing impairment.

##### Mitochondrial dysfunction

The physiological activities of the cochlea are highly susceptible to mitochondrial dysfunction. Mitochondrial dysfunction, associated with ROS, has been reported to play a central role in cochlear aging [23]. In cells, ROS is primarily derived from mitochondria and is produced in the mitochondrial oxidative respiratory chain. Disruptions in mitochondrial structure and function can lead to the accumulation of ROS. Additionally, mitochondrial DNA is a primary target of free radical attacks, and mutations in this DNA gradually accumulate with age.

###### Mitochondrial deletion mutation

The 4977 bp deletion mutation in human mitochondrial DNA, commonly referred to as the common deletion mutation, is observed not only in the tissues of patients with mitochondrial encephalomyopathy, progressive external ophthalmoplegia, and Kearns-Sayre syndrome but also in the aging tissues of elderly individuals, where it gradually accumulates with increasing age [24]. A study reported that among 17 elderly patients with presbycusis, 14 exhibited the 4977 bp deletion mutation in their inner ear tissues, whereas only 8 out of 17 individuals with normal hearing tested positive for this mutation [25]. This suggests a relatively high detection rate of the deletion mutation in elderly patients with presbycusis. Additionally, research on human temporal bone samples indicated that the prevalence of the common deletion of mitochondrial DNA in the inner ear tissues of elderly patients with presbycusis is closely associated with the degree of hearing loss [26]. In D-galactose-induced aging rat models, similar pathological changes to those observed in naturally aging rats were noted in both the peripheral and central auditory organs, accompanied by the common deletion of mitochondria. Furthermore, an increase in the 4834 bp deletion mutation of mitochondrial DNA in the inner ear of rats was found to enhance the susceptibility of inner ear tissues to aminoglycoside antibiotics and other ototoxic factors, such as high-fat diets [27, 28].

###### Mitophagy

Mitophagy is essential for cell survival and cochlear function. It refers to the selective degradation of damaged mitochondria through the autophagy pathway, which is crucial for maintaining cellular homeostasis [29]. Mitophagy serves as a significant mechanism for mitochondrial quality control and plays a vital role in the pathological changes associated with presbycusis [30]. As individuals age, the expression of mitophagy-related genes and proteins declines, leading to a reduction in mitophagy levels [31]. Studies have shown that damaged mitochondria accumulate in the cochlear hair cells and spiral neurons of aged mice, while the colocalization of autophagosomes and lysosomes decreases, indicating an impairment in mitophagy function within the central auditory system. Activating mitophagy can enhance mitochondrial degradation, slow down cellular aging, and promote the survival of cochlear hair cells [32].

##### Dysfunction of endoplasmic reticulum

Cellular stress resulting from the abnormal accumulation of unfolded or misfolded proteins in the endoplasmic reticulum (ER) is increasingly recognized as a potential contributor to various human diseases, including cancer, diabetes, obesity, and neurodegenerative disorders. The maintenance of ER protein homeostasis is regulated by the unfolded protein response (UPR), a signaling pathway that monitors the accuracy of protein folding within the ER lumen. Research has shown that with aging, the UPR-related pathway proteins, including ATF6, PERK, and IRE1, in the intermediate cells of the stria vascularis of the cochlea, exhibit upregulation. Furthermore, the upregulation of the ER chaperone protein HSP90AA1 has been found to mitigate ER stress-induced damage associated with age-related hearing loss [5].

##### Recommendations:

(1) PTA, OAE, speech recognition rate, and ABR can be used as important markers to evaluate auditory system aging, and increased values suggest impaired hearing function (Level A, Class I).
(2) MMSE and MoCA can reflect the cognitive function in auditory system aging (Level C, Class III). CAEP can be considered as a functional marker for assessing speech recognition and cognitive function in patients with presbycusis (Level B, Class I).
(3) Increasing of glutamate, ROS, IL-1β, IL-6, and TNF-α suggested disruption of cochlear function and internal cochlear environment (Level C, Class III). Mitochondrial deletion mutation, decreased mitophagy and upregulation of ER stress response proteins in the inner ear tissues may indicate cochlear physiological dysfunction (Level C, Class III).

### Structural markers of auditory system aging

Aging of the auditory system impacts several key auditory structures, including (i) an increase in middle ear impedance, a reduction in acoustic compliance, and diminished eustachian tube function; (ii) decreased energy transduction resulting from aging and atrophy of the cochlear stria vascularis, along with thickening, calcification, and hyaline degeneration of the basilar membrane, atrophy of both inner and outer hair cells, a reduction in the number of supporting cells, atrophy of the spiral ligament, degeneration of spiral ganglion cells, and degeneration of cochlear nerve fibers; and (iii) degeneration of neurons in the central auditory pathway.

#### Degeneration of structure and function of the middle ear

The middle ear undergoes changes with aging. A cross-sectional comparative study involving 103 elderly patients identified abnormalities in middle ear impedance, which were significantly associated with age-related hearing loss [33]. In another study, wideband tympanometry testing (WBT) was conducted on 58 participants with presbycusis and 52 participants with normal hearing, revealing significant differences between the two groups [34].

#### Degeneration of the organ of Corti

Age-related structural and chemical changes can occur throughout the peripheral and central auditory systems, with the cochlear organ of Corti being the structure most susceptible to age-related histopathological changes. The cells of the inner ear and the associated nerve pathways are highly differentiated. Histopathological studies of the organ of Corti have demonstrated that degeneration of sensory hair cells occurs following the loss of supporting cells. This degeneration typically begins at the basal turn, where it is most severe [35]. The loss of outer hair cells (OHC) is particularly pronounced in individuals over 70 years old, and the degeneration of inner hair cells (IHC) and OHC occurs independently. A loss of hair cells of 10 mm or more from the base can lead to significant hearing loss in the high-frequency region.

#### Degeneration of spiral ganglion cells

A series of studies have demonstrated the relationship between age and the loss of spiral ganglion neurons (SGNs) [36–38]. Young individuals possess between 30,000 and 40,000 SGNs, which declines to fewer than 20,000 by the ages of 81 to 90, indicating a progressive loss of approximately 2000 neurons every decade. The age-related loss of SGNs is most pronounced in the basal turn of the cochlea. Additionally, there is a reduction in the number of cochlear nerve fibers, with the most significant loss occurring within 10 mm of the cochlear base [39]. IHC loss is consistently associated with SGN loss; however, atrophy of afferent nerve fibers and their cell bodies can occur with aging, even in the presence of IHCs. Elderly individuals with SGN loss often exhibit poor speech recognition scores. Furthermore, neurons undergo several alterations with aging, including diminished neural synchrony, reduced neural inhibition, prolonged neural recovery time, and changes in IHC and auditory nerve synapses. Age-related modifications in inhibitory neurotransmitter levels ultimately affect the presentation of neurons in the central nervous system of the elderly.

#### Changes of stria vascularis

The stria vascularis (SV) is a key structure within the cochlea, characterized as a highly vascularized stratified epithelial tissue located on the lateral wall of the cochlea. It is externally connected to the spiral ligament of the basilar membrane and internally to the endolymph [40]. The SV maintains the ion concentration gradient essential for the mechanical–electrical signal transduction of sensory hair cells by regulating the secretion of endolymph, which is critical for generating the endocochlear potential and sustaining cochlear homeostasis [41]. As early as 1964, Schuknecht identified atrophy and dysfunction of the SV as a prevalent pathological change associated with presbycusis. When the total area of damage to the SV exceeds 30%, a flat type of hearing curve may emerge. Atrophy of the SV typically occurs in the middle and apical turns of the cochlea; however, it can also affect the basal turn due to the continuity of endolymph within the cochlear duct. These alterations result in functional impairment of the entire cochlear organ of Corti.

#### Age-related changes of brain stem and cortical areas

During the aging process of the auditory system, changes occur in the central auditory pathways and nuclei, including cell atrophy, cell loss, and a reduction in the volume of nuclei. In the cochlear nucleus (CN), the characteristics of signals—specifically time, frequency, intensity, and duration—are analyzed and transmitted to the superior olivary complex (SOC) [42]. All afferent auditory pathways form synapses in the inferior colliculus (IC), which is responsible for integrating auditory information in brainstem nuclei and transmitting it to the auditory cortex via the medial geniculate body. Age-related presynaptic and postsynaptic changes, along with functional alterations, exist in the CN, SOC, IC, and primary auditory cortex. These changes primarily include a reduction in the number, volume, and density of neurons; increased pigmentation; a decrease in the number of myelin sheaths in the ventral cochlear nucleus (VCN) of the elderly; a decline in the number of small blood vessels and capillaries per unit area with age; and an increase in lipofuscin accumulation [43]. The age-related decline in auditory nerve activity leads to the downregulation of glycinergic function in the CN, while the loss of neural synchrony contributes to diminished speech comprehension abilities in elderly individuals [44]. The characteristics of age-related central neuronal changes in the auditory system include neuronal loss, alterations in neuronal size, reductions in cell body and nucleolus size, and a reduction or disappearance of dendritic branches, or dendritic elongation. Additional functional changes in the auditory nervous system encompass dendritic morphological changes, modifications in neurotransmitter receptors, and variations in electrophysiological characteristics. Spontaneous excitatory postsynaptic currents are reduced, and the discharge patterns of neurons involved in information processing are disrupted.

##### Recommendations:

(1) The level of middle ear impedance, acoustic compliance, and eustachian tube function can reflect the degree of auditory system aging (Level C, Class IIb).
(2) Abnormity of the function and structure of the organ of Corti occurs in auditory system aging (Level C, Class III).
(3) The loss of SGN and ribbon synapses of hair cells and the change of electrophysiological activity of spiral neurons suggest degeneration of SGN during auditory system aging (Level C, Class III).
(4) Changes of the thickness of the SV and abnormal cochlear endolymphatic potentials may indicate the physiological dysfunction of the SV (Level C, Class III).
(5) The decrease of number, volume and density of neurons in the auditory cortex, the increase of pigmentation, and the decrease of myelin sheath in the ventral cochlear nucleus suggest the degradation of brain stem and cortical areas (Level C, Class III).

### Humoral markers of auditory system aging

The analysis of bioactive components in body fluids, such as blood, offers the advantages of being minimally invasive and highly sensitive, making it particularly suitable as an auxiliary indicator for the clinical assessment of auditory system aging. In the elderly population, the development of early humoral biomarkers can aid in identifying individuals at high risk of hearing loss, thus preventing the onset and progression of presbycusis. This consensus aims to recommend biomarkers associated with auditory system aging. Consequently, the screening strategy for fluid biomarkers focuses on identifying those that exhibit a strong correlation with auditory system aging in body fluids. These fluid biomarkers can provide critical supplementary information for the diagnosis and intervention of auditory system aging. However, it is important to note that these fluid biomarkers may also fluctuate in various processes of sensorineural hearing loss and are not specific indicators of auditory system aging; therefore, they should be meticulously evaluated when applied in research concerning auditory system aging.

#### Blood markers

##### Protein markers in blood

Glycated hemoglobin (HbA_1c_) is a product formed through the non-enzymatic reaction between hemoglobin in red blood cells and sugars present in the serum. It is widely utilized as a monitoring indicator for diabetes in clinical practice. Diabetes is among the most prevalent chronic diseases globally, characterized by elevated blood glucose levels resulting from defects in insulin secretion within the human body. Research has demonstrated a significant association between age-related hearing loss and the progression of diabetes [45]. Furthermore, a recent cohort study revealed that elevated fasting blood glucose and HbA_1c_ levels are positively correlated with the severity of age-related hearing loss [46], indicating that HbA_1c_ may serve as a crucial biomarker for age-related hearing loss.

Apolipoprotein J (ApoJ), also known as clusterin, is a glycosylated α-β heterodimer extracellular chaperone protein encoded by the CLU gene. It primarily exists in human high-density lipoprotein and very high-density lipoprotein and is expressed in various tissues, with the highest expression found in brain tissue, predominantly produced by astrocytes. The physiological functions of ApoJ are complex; it can remove cell debris and misfolded proteins, thereby providing cellular protection through its chaperone effect. Additionally, it functions as a cell messenger, regulating pro-apoptotic pathways, promoting lipid metabolism, modulating atherosclerosis, influencing the complement system, exhibiting anti-apoptotic properties, and facilitating cell-cell interactions [47]. ApoJ is implicated in the pathological processes of neurodegenerative diseases, such as Alzheimer’s disease. Notably, hearing loss has been identified as a risk factor for Alzheimer’s disease [48], indicating a potential correlation between the two conditions. Furthermore, a study has revealed that the expression of ApoJ is upregulated in the serum of individuals susceptible to age-related hearing loss, suggesting that ApoJ may play a role in the onset and progression of this neurodegenerative disease [49].

Thrombospondin (TSP) is a trimeric regulatory glycoprotein and an endogenous inhibitor of angiogenesis. It plays a crucial role in cellular communication, both between cells and the extracellular matrix. TSP regulates cytokines on the cell surface and is significant in the growth and differentiation of tissues and cells [50]. TSP-l, an important member of the TSP family, promotes granulocyte chemotaxis, platelet activation and aggregation, endothelial cell apoptosis, and inhibits endothelial cell proliferation. It is closely associated with the onset and progression of obesity, diabetes, and cardiovascular diseases [51]. Notably, studies have shown that the expression of TSP in the serum of patients susceptible to presbycusis is down-regulated, indicating that vascular factors may be closely linked to the development of presbycusis. Furthermore, improving vascular health may help slow the progression of this condition [49].

D-dimer is a specific degradation product of cross-linked fibrin. An increase in D-dimer levels reflects the concurrent activation of coagulation and fibrinolysis systems, making it a significant indicator of thrombosis *in vivo*. It is also valuable for distinguishing between primary and secondary fibrinolysis and for monitoring thrombolytic therapy. In clinical practice, D-dimer levels hold important diagnostic value for thrombotic diseases, evaluating thrombolytic therapy, monitoring thrombosis recurrence, and assessing cardiovascular disease and cerebral infarction [52]. Reports indicate that serum D-dimer levels significantly differ between elderly patients with sensorineural hearing loss and healthy controls, suggesting that serum D-dimer levels may serve as an important prognostic indicator for presbycusis [53].

##### Cell markers in blood

Red blood cell deformability (RCD) refers to the ability of red blood cells to alter their shape in response to external forces. This property is primarily influenced by three factors: the viscoelasticity of red blood cells, the internal viscosity of red blood cells, and their geometric configuration [54]. Superoxide dismutase (SOD) protects cells by scavenging superoxide anion free radicals, whereas malondialdehyde (MDA), a product of lipid peroxidation, can cause cross-linking of enzymes and other compounds containing amino groups, resulting in a loss of activity and subsequent damage to the structure and function of the cell membrane [55]. Plasma samples from patients with presbycusis and healthy elderly individuals were analyzed. The results indicated a significant decrease in SOD levels among patients with presbycusis, alongside a notable increase in MDA levels, which corresponded with a marked reduction in red blood cell deformability [56]. It is suggested that these changes may be attributed to the influence of external environmental factors (such as diet and environmental conditions) and internal factors (such as atherosclerosis and hypertension). Excessive free radicals can lead to the peroxidation of unsaturated fatty acids in cell membranes, particularly in red blood cells, producing MDA and other lipid peroxides. This process results in damage to the red blood cell membrane, thereby affecting its deformability and, subsequently, the microcirculation of the inner ear. This compromised microcirculation can result in a hypercoagulable state of blood, ultimately affecting the blood and oxygen supply to the inner ear. Such conditions may lead to chronic ischemia and hypoxia, culminating in sensorineural hearing loss (presbycusis).

##### Hormone-related markers in blood

A variety of hormones, including insulin-like growth factor 1 (IGF-1), estrogen (E), and aldosterone (ALD), play a significant role in auditory function. As disease and/or age progress, the expression of these hormones begins to decline sharply, ultimately impacting the cochlear structure, the integrity of cochlear cells, and the overall function of the auditory system [57].

IGF-1 is essential for the regulation of cochlear development, growth, and differentiation, with its mutations linked to hearing loss in both mice and humans. Research has shown that low levels of IGF-1 may contribute to the progression of age-related hearing loss [58]. Neural and sensory cells in the ear originate from a common progenitor cell, and their maturation necessitates cell survival, proliferation, and differentiation. During the early stages of inner ear development, the proliferation of neural progenitor cells relies on the activation of the PI3K–AKT pathway by IGF-1 to ensure survival [59]. IGF-1 can mitigate the apoptosis of cochlear hair cells and enhance their survival by reducing inflammation and oxidative stress, inhibiting the expression of pro-apoptotic genes, and regulating glucose transporters. In the elderly, the expression of IGF-1 in the inner ear gradually diminishes, leading to inflammatory responses, a failure of the cell renewal mechanism, and an acceleration of presbycusis [58]. Consequently, IGF-1 may serve as a biomarker for assessing aging in the auditory system.

Estrogen has been reported to possess neuroprotective effects and to alleviate symptoms of neurodegenerative diseases through complex cellular signaling mechanisms activated by estrogen receptors (ERs) [60]. Both human and animal studies indicate that low estrogen levels may impair hearing, potentially through mechanisms, such as altered cochlear blood flow, neuromodulation, neurophysiology, or otic capsule bone metabolism [61]. Additionally, decreased estrogen levels following menopause may be linked to hearing loss in elderly women. Hormone therapy post-menopause is believed to slow the progression of age-related hearing loss. Estrogen may delay the onset of presbycusis by enhancing antioxidant, anti-apoptotic, and anti-inflammatory effects; thus, a decline in estrogen levels is likely to have adverse effects on the inner ear environment [62].

The endocochlear potential (EP), generated by the electrical transport of potassium ions by the SV cells to the tunica media, typically ranges from 80 to 100 mV in individuals with high hearing function. Subsequent research has shown that EP values decline with age and exhibit a positive correlation with Na^+^–K^+^ pumps [63]. The level of EP is critical for auditory sensitivity and acuity, as it converts sound vibrations into electrochemical signals that are transmitted to the brain by spiral ganglion neurons (SGNs). Mice treated with ALD for 2 months demonstrated a reversal of autoimmune cochlear dysfunction, accompanied by elevated Na^+^ transport in the cochlear SV. Clinically, patients with high serum levels of ALD outperform their peers with lower levels in hearing tests [64]. These findings suggest that ALD may mitigate age-related hearing loss by regulating the Na^+^–K^+^ pump subunits α and β. Consequently, ALD may serve as a biomarker for aging in the auditory system; however, further clinical studies are necessary to evaluate its practical utility.

##### miRNA-related markers in blood

microRNAs (miRNAs) are a class of highly conserved, single-stranded non-coding RNAs that range in length from 19 to 25 nucleotides. Numerous studies have demonstrated that miRNAs play a significant role in biological development, cell differentiation, and the regulation of target genes associated with diseases. They are fundamental to gene regulation in nearly all multicellular organisms, including the modulation of aging processes [65]. Currently, many miRNAs have been implicated in age-related hearing loss, as they influence the aging of the auditory system through various signaling pathways, including insulin/IGF-1 signaling, the mTOR pathway, translation signaling, deacetylase activity, mitochondrial/reactive oxygen species signaling, and the DNA damage response [66].

The miR-183 family gene cluster is highly conserved across vertebrates and invertebrates, comprising miR-183, miR-96, and miR-182, which are expressed with notable specificity in sensory neurons and sensory epithelial cells. Investigation of the expression patterns of miR-183, miR-96, and miR-182 in the developing inner ear revealed that polycistronic clusters of these three miRNAs are abundantly expressed in afferent cochlear and vestibular neurons, as well as their peripheral nerve targets, specifically auditory and vestibular hair cells [67]. The dynamic changes in expression during inner ear development, along with their evident expression during hair cell differentiation in Balb/c mice, suggest that these miRNAs play a crucial role in hair cell differentiation and maturation. Although loss of function of the miR-183/96/182 cluster did not impede hair cell production, it resulted in several defects in hair cell development and function: (i) misalignment of hair cells in the cochlear epithelium; (ii) absence of epidermal plate formation; (iii) loss of mechanoelectrical transduction function; and (iv) complete degeneration of hair cells, leading to severe hearing loss in the experimental mice by postnatal day 18 [68].

miR-34 is a highly conserved miRNA, characterized by a homologous seed sequence found across various organisms, including flies, nematodes, mice, and humans. The miR-34 family comprises three members: miR-34a, miR-34b, and miR-34c. Research indicates that levels of miR-34a in the cochlea, auditory cortex, and plasma consistently increase with aging in C57BL/6 mice [69]. These elevations correlate with heightened hearing thresholds and significant loss of hair cells. Furthermore, plasma miR-34a levels were notably higher in patients with presbycusis compared to controls with normal hearing. Consequently, the level of miR-34a may serve as an effective biomarker for the early detection of presbycusis. Pharmacological inhibition of the miR-34a/Sirt1 signaling pathway has been shown to enhance mitotic phagocytosis, promote mitochondrial biosynthesis, and reduce oxidative stress-induced cell death in the organ of Corti [70]. Both overexpression of Sirt1 and deficiency of miR-34a mitigated age-related cochlear hair cell loss and hearing impairment in mice.

Compared to young C57BL/6 mice, cochlear hair cells in aged C57BL/6 mice exhibited significant degeneration, accompanied by elevated expression of miR-29b and an age-related down-regulation of Sirt1 and Pgc-1α [71]. In HEI-OC1 cells, the overexpression of miR-29b inhibited the expression of Sirt1 and Pgc-1α, leading to mitochondrial dysfunction and increased apoptosis. Conversely, the down-regulation of miR-29b and the up-regulation of Sirt1/Pgc-1α can inhibit the apoptosis of cochlear hair cells, while the down-regulation of Sirt1/Pgc-1α may increase the incidence of presbycusis by promoting the apoptosis of cochlear hair cells.

#### Urine markers

Neopterin is recognized as an inflammatory metabolite. A longitudinal cohort study assessed baseline levels of inflammation by measuring neopterin levels in monthly urine samples from 45 independent older adults aged 65 to 75 years. Participants were classified into various risk categories based on the frequency of neopterin elevation observed over a 12-month period. Hearing was evaluated using pure-tone audiometry at baseline, as well as at 1 year and 3 years. The results indicated that individuals in the highest risk category, where neopterin levels improved more than 50% of the time, experienced greater deterioration in hearing, particularly in high-frequency ranges [72]. Consequently, neopterin may be considered a potential biomarker for assessing the aging of the auditory system.

The level of oxidative stress within the auditory system plays a significant role in presbycusis. Auditory oxidative stress levels can be indicative of systemic oxidative stress. Systemic oxidative stress can be evaluated by measuring the urinary content of metabolites derived from organic compounds. Research has shown that the urinary metabolite glutathione-dependent thioglycolic acid level can serve as a reflection of susceptibility to oxidative stress-induced hearing loss [73]. Additionally, another study has demonstrated that cocoa can elevate the concentration of polyphenols in urine, thereby enhancing total antioxidant capacity and offering protection against age-related hearing loss [74]. It is proposed that urinary polyphenol content may also be utilized as a marker for assessing the aging of the auditory system.

##### Recommendations:

(1) Abnormal blood protein levels (HbA_1C_, ApoJ, TSP, D-dimer), red blood cell degeneration, hormone levels (IGF-1, Estrogen, ALD) and miRNA levels (miR-183, miR-34, miR-29) can indicate the aging process of the auditory system (Level C, Class IIb).
(2) Increased levels of inflammatory urine metabolites such as neopterin, glutathione-dependent thioglycolic acid and polyphenol may indicate cochlear physiological dysfunction in auditory system aging (Level C, Class IIb).

## Construction of "ear age" prediction models

### Data collection

#### Sample selection

(1) People of different ages, genders, and living environments were selected as the research objects to ensure the diversity and representativeness of the samples.
(2) Samples including normal hearing people and people with different degrees of hearing loss.

#### Data collection

(1) A comprehensive ear examination, including pure tone audiometry, otoacoustic emission, auditory brainstem response, etc., was performed to evaluate the hearing level.
(2) Biological samples such as blood and urine were collected to detect the levels of aging markers of the auditory system.
(3) Information on lifestyle, medical history, and family history were collected as potential influencing factors.

### Model establishment

#### Selection of modeling methods

(1) Machine learning algorithms such as multiple linear regression, support vector machine and random forest can be used.
(2) Considering that there may be a nonlinear relationship between auditory system aging markers and ear age, some nonlinear modeling methods may be more appropriate.

#### Variable selection

(1) In addition to the markers of auditory system aging, age, gender, history of noise exposure, history of ear diseases and other factors can also be included in the model.
(2) Stepwise regression, principal component analysis and other methods were used to select variables to remove redundant variables and improve the accuracy and stability of the model.

#### Model training

(1) The collected data were used to train the model and adjust the model parameters so that the model could accurately predict ear age.
(2) The data can be divided into training set and test set for cross-validation to evaluate the performance of the model.

### Model evaluation and optimization

#### Evaluation indicators

(1) The commonly used evaluation indexes include mean square error, mean absolute error, coefficient of determination, etc.
(2) The accuracy, sensitivity, specificity, and other indicators of the model can also be calculated to evaluate the predictive ability of the model for different ear age ranges.

#### Model optimization

(1) According to the evaluation results, the model was optimized. The model parameters can be adjusted, new variables can be added, and modeling methods can be improved.
(2) The process of model training and evaluation was repeated until satisfactory performance was achieved.

### Model application

#### Application model

The established ear age prediction model is applied to clinical practice and scientific research to provide a basis for ear health assessment, hearing protection, and intervention.

#### Validation model

(1) New sample data were collected to verify the model to ensure the stability and generalization ability of the model.
(2) Update and improve the model as time goes by and new data accumulate.

By constructing an ear age prediction model including aging markers of the auditory system, the degree of ear aging of individuals can be more accurately evaluated, which provides strong support for early detection of hearing problems and the development of personalized hearing protection strategies.

## Conclusion and future perspectives

According to the expert discussion, the auditory system aging markers are recommended in the three dimensions, including function, structure, and body fluids. These markers will be further validated in different age groups in the future (Table 3).

**Table 3.** Recommended biomarkers of auditory system aging

Dimension	Biomarker	Test method	COR	LOE
Functional markers	PTA, ABR, SOAE, EOAE, Pbmax and P-I curve	Hearing detect device	I	A
	MMSE and MoCA	Questions and answers	III	C
	CAEP	Electrophysiology	I	B
	mtDNA, mitophagy, UPR	Cochlea/PCR/WB	III	C
	Glutamate, ROS, IL-6, IL-1β, TNF-α	Cochlear endolymph/ELISA	III	C
Structural markers	WBT	Acoustic immittance measurement	IIb	C
	IHCs and OHCs	Cochlea/HE	III	C
	SVs and SGNs	Cochlea/HE	III	C
	CN, SOC, IC, and VCN	Brain/IF	III	C
Humoral markers	HbA_1c_, ApoJ, TSP, D-dimer, and RCD	Plasma/ELISA/ viscometry	IIb	C
	IGF-1, E, ALD, and miRNAs	Plasma/ELISA/PCR	IIb	C
	Neopterin	Urine/UPLC–MS	IIb	C

Abbreviations: ABR, auditory brainstem response; ALD, aldosterone; ApoJ, apolipoprotein J; CAEP, cortical auditory evoked potential; CN, cochlear nucleus; E, estrogen; ELISA, enzyme-linked immunosorbent assay; EOAE, evoked otoacoustic emission; HbA_1c_, glycosylated hemoglobin; IC, inferior colliculus; HE, hematoxylin and eosin staining; IF, immunofluorescence; IGF-1, insulin-like growth factor 1; IHCs, inner hair cells; IL-1β, interleukin 1β; MMSE, mini mental status exam; MoCA, montreal cognitive assessment; mtDNA, mitochondrial DNA; OHCs, outer hair cells; Pbmax, maximum speech recognition score; PCR, polymerase chain reaction; PTA, pure tone audiometry; RCD, red blood cell deformability; ROS, reactive oxygen species; SGNs, spiral ganglion cells; SOAE, spontaneous otoacoustic emission; SOC, superior olivary complex; SVs, stria vascularis; TNF-α, tumor necrosis factor α; TSP, thrombospondin; UPLC-MS, ultra-high performance liquid chromatography-tandem mass spectrometry; UPR, unfolded protein response; VCN, ventral cochlear nucleus; WB, western blotting; WBT, wideband tympanometry test.

With the continuous development of biotechnology and data analysis methods, the construction of ear age prediction models will be more accurate and efficient. The following aspects can be further explored in the future: (i) Integration of multi-omics data: Combining genomics, transcriptomics, proteomics, and other multi-omics data to comprehensively reveal the molecular mechanism of auditory system aging. (ii) Application of big data technology: using big data technology to mine and analyze massive data to find new auditory system aging markers and predictors. (iii) Construction of personalized prediction model: To construct a personalized ear age prediction model based on individual genetic background, living environment, and other factors to improve the accuracy of prediction.

In conclusion, the construction of ear age prediction model is a research field involving multi-disciplinary intersection, which needs continuous exploration and innovation. By constructing accurate and reliable ear age prediction models, we can better understand the mechanism and law of auditory system aging, and provide the scientific basis for the prevention and treatment of age-related hearing loss and other diseases.
